# Supplementary material for: Paralogous synthetic lethality underlies genetic dependencies of the cancer-mutated gene STAG2
Source: Life Sci Alliance. 2021 Aug 30;4(11):e202101083. doi: 10.26508/lsa.202101083 (PMC8408347; doi:10.26508/lsa.202101083)
Supplement: Supplementary file 1 [file LSA-2021-01083_TableS1.docx]

Table S1- Knockout cell lines generated for this paper

| Cell Line Name | sgRNA introduced | gDNA mutation | Predicted protein mutation |
| --- | --- | --- | --- |
| HAP1 *STAG2* KO c1 | sgSTAG2-1 | c.658_659insT | p.(L221Pfs*18) |
| HAP1 *STAG2* KO c2 | sgSTAG2-1 | c.658_659insA | p.(T220Nfs*19) |
| HAP1 *STAG2* KO c3 | sgSTAG2-2 | c.1614delC | p.(V539Wfs*37) |
| RPE1 *STAG2* KO c1 | sgSTAG2-1 | Allele 1: c.648_658del  Allele 2: c.659_663del | Allele 1: p.(H217Pfs*18)  Allele 2: p.(T220Sfs*17) |
| RPE1 *STAG2* KO c2 | sgSTAG2-2 | Allele 1: c.1614_1615insTAGAAATAGCAAGTTAAAATA  AGGCTAGTCCGTTTTTAGCGCCCCCTGCAGGCAGCTGCGCGCTCGCTCGCTCACTGAGGCCGCCCGGGCAAAGCCCGCC  Allele 2: c.1614_1615insC | Allele 1:  p.(V539*)  Allele 2: p.(V539Wfs*37) |
| RPE1 *STAG2* KO c3 | sgSTAG2-2 | c.1614delC | p.(V539Wfs*37) |
| HAP1 *STAG1* KO c1 | sgSTAG1-1 | c.1021_1022insG | p.(D341Gfs*21) |
| HAP1 *STAG1* KO c2 | sgSTAG1-2 | c.2014_2015insT | p.(S672Ffs*13) |
