# Supplementary material for: Paralogous synthetic lethality underlies genetic dependencies of the cancer-mutated gene STAG2
Source: Life Sci Alliance. 2021 Aug 30;4(11):e202101083. doi: 10.26508/lsa.202101083 (PMC8408347; doi:10.26508/lsa.202101083)
Supplement: Supplementary file 6 [file LSA-2021-01083_TableS6.docx]

Table S6- sgRNA sequences used for CRISPR KO and CRISPRi

| Name | Sequence | Vector |
| --- | --- | --- |
| sgSTAG2-1 | ATTTCGACATACAAGCACCC | pSpCas9-T2A-Blast; pSpCas9-T2A-puro |
| sgSTAG2-2 | ACGGGAGGATGACATTCAGC | pSpCas9-T2A-Blast |
| sgNT | GCCTTGGCTAAACCGCTCCC | pCRISPRia |
| sgSTAG1 KD #1 | GGCGGGGGCCGCGCCTTCAA | pCRISPRia |
| sgSTAG1 KD #2 | GAAGTCTGCAGCGGCGCCAT | pCRISPRia |
| sgLUC-1 | ACAACTTTACCGACCGCGCC | LentiCRISPR v2 |
| sgLUC-2 | ATAAATAACGCGCCCAACAC | LentiCRISPR v2 |
| sgIREB2-1 | AATTCGGCAGAAATCGAGAG | LentiCRISPR v2 |
| sgIREB2-2 | GATCTGTCGGACAAGCAGGA | LentiCRISPR v2 |
| sgSTAG1-1 | GGCTGGACTCTTCATGACA | LentiCRISPR v2; pSpCas9-T2A-Blast |
| sgSTAG1-2 | GATCGATTCAATCATTCTG | pSpCas9-T2A-Blast |
| sgMAU2-1 | GCAGGCAGTGCACGCACAGG | LentiCRISPR v2; pSpCas9-T2A-Blast |
| sgMAU2-2 | GCGCTGTTCCTCCTCAGCAA | LentiCRISPR v2 |
| sgNIPBL-1 | ACACAGGCATGACAATAGGA | LentiCRISPR v2 |
| sgNIPBL-2 | AAGTGAGGACTACCTACACA | LentiCRISPR v2 |
| sgPAGR1-1 | AGCCCGGAGCCAGAAACGGG | LentiCRISPR v2 |
| sgPAGR1-2 | TCCCAGACCACACATGCCCA | LentiCRISPR v2 |
| sgLACZ-1 | CGCCCGGTGCAGTATGAAGG | LentiCRISPR v2 |
